# The First Dental Work Published in the United States

**Published:** 1905-06

**Authors:** William H. Trueman

**Affiliations:** Philadelphia


### THE FIRST DENTAL WORK PUBLISHED IN THE UNITED STATES.

BY WILLIAM H. TRUEMAN, D.D.S., PHILADELPHIA.

DENTAL bibliographers assign to a little pamphlet now before me the credit of being the first dental work published in the United States, and to its author, the credit of being the first of a long line of American contributors to dental literature, whose combined work has done much to advance and ennoble the science. It is entitled:

A
TREATISE
on the
HUMAN TEETH,
concisely explaining their structure,
and the cause of
DISEASE AND DECAY:
To which is added,
The most beneficial and effectual method of
treating all disorders incident to the
teeth and gums; with directions for
their judicious extraction, and
proper mode of preservation:
interspersed with observations interesting to, and
worthy the attention of every individual.
BY R. C. SKINNER,
Surgeon Dentist

New-York
Printed by Johnson and Stryker, No. 29 Gold-Street,
For the Author.
----o----
1801
Copy-Right secured

It is a pamphlet of twenty-six octavo pages, written, as were many like publications before and since, as an advertisement of its author. Inasmuch as they contain much information the public should know, and have undoubtedly been the means of bringing to the notice of many the resources of dental science for arresting dental disorders and repairing the injury they have done, such works are commendable. Although, strictly speaking, an advertisement, this little work is well within recognized ethical lines; subsequent American writers have no cause to be ashamed of this initial work, nor of its author, so far as this work is concerned, as the father of American dental literature.

This work is exceedingly rare, a brief *résumé* of its contents may, on that account, be of interest.

The author, in his preface, says,—

"Whatever are the merits or defects of this little production, the importance of the subject treated of, as respects every individual, it is presumed, will not be denied. The author has endeavored to combine perspicuity with utility, concisely explaining the causes of disease and decay of the human teeth, their remedies, and only sure and certain method of preservation, etc., etc. The eminent writers on these interesting subjects are too voluminous and expensive to obtain general circulation. The humble efforts of the author of this little tract obviates that difficulty. It is put into the hands of the public for the inconsiderable sum of thirty cents."

His brief descriptions of the structure of the teeth, of the eruption of teeth; of their disorders and the general causes of dental decay; of the alveoli or sockets; of scurvy in the gums; of abscesses in the sockets or gums; of the tartar and septic acid; and directions for extracting teeth, are in accord with accepted ideas current at the time he wrote.

His advice to parents and guardians of children regarding the teeth of those under their charge, while brief, is to the point. He speaks of the distress which frequently accompanies the eruption of the wisdom-teeth, owing to want of room in the jaws, which he says, "must be borne a considerable time, or the tooth extracted to obtain ease." He strongly advises "lacerating or cutting the gum down to the teeth," to relieve the disorders attending difficult dentition of infants, and combats the idea that the resulting cicatrix will later occasion a more difficult eruption of the teeth. He concludes his admonitions enforcing the importance of cleanliness of the teeth and their surroundings in the following words: "It is an established principle from time immemorial, among the ancients as well as moderns, that *cleanliness contributes to health.* If this theory is admitted (which it is presumed no person will deny), it incontestably proves that its application to the teeth and gums, as constituent parts of the body, is as necessary as to the face, hands, feet, or trunk." Caries, he says, "may arise from either internal or external causes and may be divided into soft and dry." This was accepted theory at that time. He further says, "In all cases where a decay is perceptible, the rotten part should be thoroughly and judiciously removed, and the cavity perfectly and solidly filled with gold-, silver-, or lead-foil, prepared for that purpose. If the decay has penetrated to the nerve of the tooth, and pain ensues, it must nevertheless be thoroughly removed, the nerve effectually destroyed, and the cavity filled as before mentioned, or the tooth extracted; otherwise acid and saline particles will enter the hole or cavity of the tooth originally filled with the nerve or cord, wound its membranes, and probably produce an abscess in the socket and gum."

Diseases, he says, "sometimes arise in the sockets when the teeth are perfectly sound; these proceed either from a constitutional cause, or a natural effect taking place prematurely. The former may be removed by proper corrective prescriptions, frequent scarifying, or bleeding the gums, and externally applying antiscorbutic and astringent medicines. The latter is seldom, perhaps never, cured. It generally occasions a total loss of the teeth contiguous to the diseased part. This disease begins by a wasting of the alveolar processes at the edges of the socket, which gradually proceeds to the bottom; the gum loses its connection with the alveolar process and the neck of the tooth, assumes a livid appearance, and continually discharges pus from the diseased surfaces; the teeth affected at length become extremely loose, and at last drop out."

Tartar he divides into three varieties,—"the yellow, the black and the green. The two former are nearly similar in their consistence and effects; the latter is chemically denominated the septic acid. . . . The green tartar (or septic acid) is materially different from the yellow and black, both in its appearance and effects upon the teeth; this substance never forms a concretion. It first appears as a green stain upon that part of the teeth next the gum, where the enamel is very thin, gradually increasing (unless checked by art) until it nearly covers the anterior part of the incisors, canines and small molars of the maxillary superior. The collection of this substance upon the large molars is partial in proportion to what is frequently found upon the teeth before mentioned." This green tartar he credits with being rapidly destructive to the enamel of the teeth, and credits its origin to a putrefactive decomposition of food and other débris which collects about the teeth, stating that it possesses qualities nearly resembling aqua fortis.

He concludes his treatise with a short chapter upon extracting teeth, and from his directions one would infer that he used forceps for this operation. He strongly advocates saving the teeth. He says, " A tooth with one fang or root, that gives pain from any other cause than an abscess, can easily be cured and rendered serviceable, oftentimes, during a person's life. It would be bad practice to extract a front tooth or stump; because it would remove and destroy the solid base, indispensably necessary for the firm security and masticating use of an artificial tooth, which may be set to very great advantage where there is a solid stump standing."

His kindly disposition is evidenced by the following paragraph:

" Poor people afflicted with complaints in the teeth and gums, will be attended to at the dispensary, hospital, almshouse, or at the house of the operator, No. 64 Fair Street, and relieved *gratis.* A request from any physician or surgeon of this city, or any of the superintendents, trustees, or official visitors of either of those benevolent institutions, will be immediately attended to, and assistance given free of any expense."

This kind offer is duly acknowledged by the Board of Managers of the Dispensary of the City of New York, under date of September 2, 1792.

I infer, from a perusal of the following, that the author did not confine his labors exclusively to dental surgery.

ESTABLISHED FEES.

For setting an artificial eye, nose, or ear,—3 guineas each.

For setting an artificial flexible leg, perfectly to imitate nature in muscular motion—4 guineas each.

For setting a common cork leg—3 guineas.

*Transplanting* a tooth which grows firm in the head—3 guineas each.

*Grafting,* or setting human teeth in any way on gold—4 dollars each.

*Grafting,* or setting human teeth on silver—3 dollars each.

Fixing and setting best artificial teeth, on gold springs or pivots—2 dolls. 50 cents.

Grafting or setting (any way) artificial teeth of second quality—2 dollars each.

Grafting or setting third quality—1 doll. 50 cents.

Grafting or setting fourth quality—1 dollar.

Filing, eradicating caries (or rotten parts), or filling cavity with silver- or lead-foil—50 cents each.

Filling cavity with gold—1 dollar each.

Extracting teeth abroad—1 dollar each.

For extracting teeth at his own house—50 cents each. Children's teeth half price.

Eradicating tartar, and cleaning the teeth, from 1 to 3 dollars each set, in proportion to their situation.

In concluding, he says,—

"R. C. Skinner embraces this opportunity of acknowledging the very great obligation he feels himself under to several medical gentlemen of this city, who have particularly honored him with their patronage. He presents them (and every other person who has either patronized or employed him) the warmest effusions of a grateful heart; a heart that will ever feel, and acknowledge (while its pulsations continue) every obligation and favor, either from individuals or the public."

"The public are further respectfully informed, that in any and every case, where part, or the whole of the teeth are decayed and lost new ones may be substituted or set, even if there is neither tooth nor stump standing in the head, from a single tooth to a complete whole set. Even poor people may enjoy the luxury of possessing a good set of front teeth, as some are set as low as one dollar each.

"New York, June 20, 1801."

Taking it all in all, this unpretentious little pamphlet by Mr. Richard C. Skinner, Surgeon Dentist, of New York, pioneer dental writer of the United States, well holds its own when compared with the best of its class and time.

